# A Microfluidic Split-Flow Technology for Product Characterization in Continuous Low-Volume Nanoparticle Synthesis

**DOI:** 10.3390/mi10030179

**Published:** 2019-03-09

**Authors:** Holger Bolze, Peer Erfle, Juliane Riewe, Heike Bunjes, Andreas Dietzel, Thomas P. Burg

**Affiliations:** 1 Max Planck Institute for Biophysical Chemistry, Göttingen 37077, Germany; holger.bolze@mpibpc.mpg.de; 2 Center of Pharmaceutical Engineering, Technische Universität Braunschweig, Braunschweig 38106, Germany; p.erfle@tu-bs.de (P.E.); j.riewe@tu-braunschweig.de (J.R.); heike.bunjes@tu-braunschweig.de (H.B.); a.dietzel@tu-braunschweig.de (A.D.); 3 Institute of Microtechnology, Technische Universität Braunschweig, Braunschweig 38124, Germany; 4 Institut für Pharmazeutische Technologie, Technische Universität Braunschweig, Braunschweig 38106, Germany; 5 Department of Electrical Engineering and Information Technology, Technische Universität Darmstadt, 64283 Darmstadt, Germany

**Keywords:** lipid nanoparticles, online analysis, microfluidics, plug flow mixer, fluorescence, precipitation, single particle analysis, nanoparticle characterization

## Abstract

A key aspect of microfluidic processes is their ability to perform chemical reactions in small volumes under continuous flow. However, a continuous process requires stable reagent flow over a prolonged period. This can be challenging in microfluidic systems, as bubbles or particles easily block or alter the flow. Online analysis of the product stream can alleviate this problem by providing a feedback signal. When this signal exceeds a pre-defined range, the process can be re-adjusted or interrupted to prevent contamination. Here we demonstrate the feasibility of this concept by implementing a microfluidic detector downstream of a segmented-flow system for the synthesis of lipid nanoparticles. To match the flow rate through the detector to the measurement bandwidth independent of the synthesis requirements, a small stream is sidelined from the original product stream and routed through a measuring channel with 2 × 2 µm cross-section. The small size of the measuring channel prevents the entry of air plugs, which are inherent to our segmented flow synthesis device. Nanoparticles passing through the small channel were detected and characterized by quantitative fluorescence measurements. With this setup, we were able to count single nanoparticles. This way, we were able to detect changes in the particle synthesis affecting the size, concentration, or velocity of the particles in suspension. We envision that the flow-splitting scheme demonstrated here can be transferred to detection methods other than fluorescence for continuous monitoring and feedback control of microfluidic nanoparticle synthesis.

## 1. Introduction

Continuous processes provide numerous advantages over batch processes for the production of chemical and pharmaceutical products [1,2,3]. For example, continuous processes often result in significantly lower waste production [4] and better process control than batch processes [5]. Importantly, continuous processes are often amenable to a microfluidic implementation, enabling efficient production of small batches [2,3]. Several microfluidic systems for the continuous production of nanoparticles have been demonstrated in the past. Examples include systems for the synthesis of quantum dots [6,7], metal particles [8,9,10], metal oxide particles [11], drug nanoparticles [12], and polymer nanoparticles for medical application [13,14]. Liposomes [15] and lipid nanoparticles [16] have raised great interest as drug carriers. Lipid nanoparticles are of special interest for encapsulating poorly water-soluble active ingredients to facilitate their transport in the bloodstream and uptake into cells [17,18]. Since—especially at early stages of drug discovery and development—drugs are often produced only in small amounts, there is an interest in continuous microfluidic processes for synthesizing lipid nanoparticles continuously and efficiently at a small scale. By this motivation, the synthesis of lipid particles in microsystems has been demonstrated previously [19,20,21,22,23,24].

Process stability over several hours or days is an important challenge for the synthesis of uniformly sized nanoparticles in microfluidic systems. During the long time of production, the process can be altered by time-dependent changes, including mechanical wear of the pumping system, a change in the reactant composition, or the blockage of microfluidic channels by particles and gas bubbles. Such changes can compromise the quality or make the product unusable. Therefore, being able to monitor the process output is of great interest for designing reliable and robust microfluidic systems for the synthesis of nanoparticles. In contrast to an end-point analysis of small batches, online analysis allows the continuous measurement of important product characteristics and the ability to counteract any change. Adding such an analysis to a microfluidic system is tied to special requirements. First, a low volume without large cavities or dead volume is critical to avoid back mixing and to minimize the time delay between synthesis and characterization. Second, the flow rate through the detector needs to be matched to the detection bandwidth. This is important in order to ensure that particles pass at an adequate frequency while spending sufficient time in the detector to elicit a quantifiable response.

To analyze particles in a microfluidic setup, different kinds of detectors have been proposed [25,26]. The most commonly used techniques for analyzing nanoparticles are bulk methods, which analyze the collective effect induced by all particles in a reference volume. Examples of such techniques include dynamic light scattering [27,28], fluorescence spectroscopy [7,29], ultraviolet–visible (UV/Vis) spectroscopy [30], and X-ray absorption [31,32]. The drawback of bulk analysis techniques is a limited capability to detect samples with high polydispersity or a multimodal distribution. Another way to detect particles is by single particle analysis (SPA). Among the most well-known SPA-based systems are flow cytometers and cell sorters [33]. SPA detectors in microfluidic systems can use different detection principles like impedance detection [34,35,36,37,38], static light scattering [39,40,41,42,43], and fluorescence-based detection [43,44,45,46,47,48]. Often two or three of these detection principles are coupled [34,43,47,49,50]. Most microfluidic single-particle detectors described in the literature have been developed to detect and differentiate particles bigger than 1 µm. Smaller particles are significantly more difficult to detect, as the signal strength in all the above methods decreases nonlinearly with size [35,36,40,41,42,43,44,46,48,51,52].

Therefore, to achieve an adequate signal-to-noise ratio, it is important to choose the flow rate through the detector slow enough to be able to measure at a relatively low bandwidth. However, the direct sequential coupling of continuous-flow synthesis with a flow-through detector does not allow independent flow-rate adjustment. Therefore, while nanoparticles have been measured in microfluidic systems by different bulk sensors online [6,7,27,28,30,31,32], there are no studies, to our knowledge, combining a low sample volume microfluidic online SPA detector for sub-micron particles with a nanoparticle synthesis setup.

In this study, we combine a microfluidic lipid nanoparticle precipitation setup with a fluorescence detection system able to detect the nanoparticles in a small stream. In our system, lipid nanoparticles are synthesized by solvent-antisolvent-precipitation, in which an organic phase containing the lipid and a fluorescent dye is mixed with an excess of water [53]. The organic solvent, which is miscible with water, diffuses into the much bigger water volume. In the mixture, the character of the water dominates so that the lipids precipitate, encapsulating the dye. Since a higher oversaturation leads to a reduced particle size, rapid mixing to homogeneity is important to avoid concentration gradients that would result in a broad size distribution. To achieve this, we employed a plug-flow mixer first described by Erfle et al. [24], in which the fluids that are to be mixed are combined in plugs divided by gas. As these plugs propagate through the system, rapid mixing takes place due to recirculation of the fluids within the plugs.

The detector uses a micron-sized channel instead of a focusing sheath stream to ensure the measurement of single particles in a defined detection spot [48,51,54]. The whole particle suspension is flown through a microfluidic channel. A much smaller measurement channel branches off this channel. The small profile of the measurement channel (2 µm width and height) confines the volume of liquid and ensures that a great majority of the detection events is caused by isolated single particles. Detection itself is accomplished by focusing a laser on a part of the small channel to excite the fluorescent dye Nile Red, which is added to the lipid nanoparticles during precipitation. A characteristic phenomenon of Nile Red is that its fluorescence is heavily quenched in water so that the accumulated lipophilic dye molecules in the lipid particles are much brighter than the ones remaining in water [55]. By the combination of the quenching and the lipophilic accumulation in the lipid particles, the particles generate a fluorescent intensity much stronger than the surrounding liquid. The fluorescence emission is collected and analyzed. Here each passing particle generates a peak, the height of which corresponds to the number of fluorophores in the particle. The length of the peaks corresponds to the residence time in the detection spot. Assuming homogenous distribution of fluorophores in the lipid volume, the peak height can be used as a measure for particle size, while the residence time allows the measurement of the velocity in the channel. Based on the velocity, geometrical parameters and the number of particles per minute, the concentration of particles can be measured.

## 2. Materials and Methods

### 2.1. Mixing Chip

The mixing system included a micromixer which operated according to the principle of segmented gas–liquid flow [24]. Borosilicate glass (Schott BOROFLOAT^®^ 33, Schott AG, Mainz, Germany) was chosen as the material because it is mechanically and chemically stable, biocompatible, and transparent for optical observations [12,56,57]. The fabrication of the microsystem and the structuring of the glass was performed by femtosecond laser ablation (microSTRUCT c; 3D Micromac AG, Chemnitz, Germany). Details about the manufacturing process can be found in Erfle et al. [24]. The micromixer has a symmetrical design in which the solutions are divided after entry and reunited at the flow-focusing point (see Appendix A). By this arrangement, the organic phase was focused in the middle of the channel, and the contact to the wall was limited to prevent fouling. The streams were generated by two syringe pumps (neMESYS 290N, Cetoni GmbH, Korbussen, Germany). Due to the small dimensions, mixing by convection was limited. To increase the mixing efficiency, nitrogen was injected at the flow-focusing point where the streams merge, achieving immediate segmentation of the continuous flow into liquid plugs. In these liquid plugs, recirculation was induced, which increases the mixing rate. In the mixture, the oversaturation of solvent led to the precipitation of particles, so a higher oversaturation increased the number of small particles. Thus, better mixing quality led to a smaller and more homogeneous size of the precipitated particles. The gas stream was regulated by a pressure regulator (OB1 MK3, ELVESYS, Paris, France), which allowed to control the plug length and thereby influence the particle size. A camera was used to measure the plug size and allowed adjustment of the particle/plug size.

**Figure 1:**
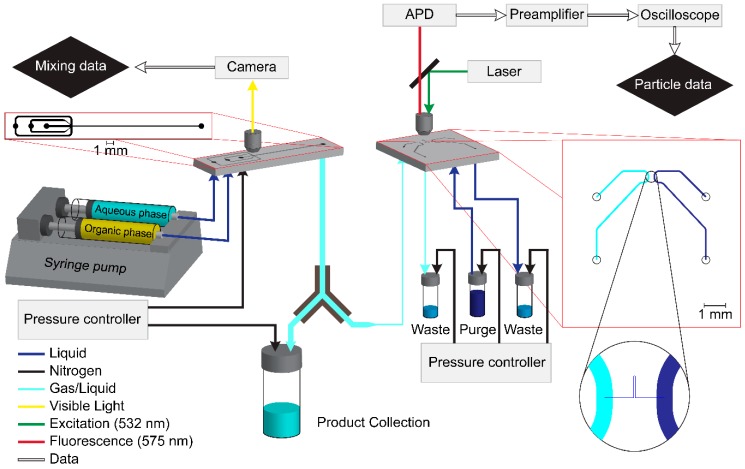
Flowchart and structure of the online analysis setup.

The mixing chip was linked to the detector by capillary tubing made of polyether ether ketone (PEEK) with an inner diameter of 0.5 mm and an outer diameter of 0.821 mm with a length between 5 and 10 cm (Figure 1). The major part of the product stream was routed to a pressurized vial without passing the detector. A fraction of the product stream was rerouted by a Y-connector to the detector. The vial was pressurized up to 0.8 bar to drive the liquid through the detector.

### 2.2. Detection Setup

The particle detection chip consisted of a structured silicon element closed by Borofloat glass. The chips were fabricated using lithography and standard silicon processing. The small measurement channel was first etched into silicon anisotropically by deep reactive ion etching (DRIE) on an STS Multiplex system (Surface Technology Systems, Newport, UK) using a Bosch process with alternating cycles of C4F8 for sidewall passivation and SF6 for etching. Large supply channels were then patterned by lithography and etched to a depth of 30 µm using a Bosch process. After this step, the channels were sealed by anodic bonding to an unstructured Borofloat wafer, and access holes were etched through the silicon from the backside using DRIE. The channel structure contained two 75 µm wide supply channels, which were connected by a measurement channel only 2 µm deep and wide. These dimensions were the design values. An example of a real device cross section is shown in the appendix.

The chip was connected to capillary tubing by a special holder allowing a microscope objective to operate at a distance less than 2 mm from the glass surface and, at the same time, connect the capillaries tightly to the backside of the chip. The buffer supply channel was filled with Hellmanex solution to purge stuck particles from the chip every four minutes. The outlet of the product supply channel was connected to a pressure-controlled vial. Additional pressure-controlled vials were connected to the inlet and outlet of the buffer supply channel. These pressure-controlled vials provided a tunable and stable flow, driving the particle suspension through the measurement channel or fresh buffer solution through the purge channel.

The analyzed channel area was illuminated by a 532 nm laser (CPS 532, Thorlabs, Newton, NJ, USA), which was focused on the chip by the microscope objective (LD-Plan NEOFLUAR 40×/0.6 Corr Ph2, Carl Zeiss AG, Oberkochen, Germany). The same objective collected the emitted light and projected it on a detector after passing a dichroic mirror (MD 568, Thorlabs, Newton, NJ, USA) and an emission filter (FELH0550, Thorlabs, Newton, NJ, USA). The light was analyzed by an avalanche photodiode (APD 110A, Thorlabs, Newton, NJ, USA). The signal of the photodiode was amplified 200 times and low-pass filtered at 10 kHz by a preamplifier (SR 560, Stanford Research Systems, Sunnyvale, CA, USA) before it was analyzed by an oscilloscope (PicoScope 4262, Cambridgeshire, UK). The oscilloscope measured the signal every 10 µs in a range of 5 V (DC).

For experiments using reference particles to characterize the whole system, the injected organic phase was replaced by an aqueous solution of reference particles, while all other solutions, pressures, and apparatus stayed the same to allow the characterization of parameters without changing concentration.

### 2.3. Chemicals and Solutions

The detector was calibrated and characterized using an aqueous solution containing fluorescent beads with a nominal diameter of 200 nm (F-8809, Thermo Fisher Scientific, Waltham, MA, USA) at a concentration of 4 * 10^6^ particles per milliliter. In addition, the solution contained Rhodamine to detect the flow direction even in case of clogging (10 µg/mL; Quality for Fluorescence, Merck, Darmstadt, Germany), sodium chloride (5.48 mg/mL, >99.8%, Carl Roth, Karlsruhe, Germany), sodium azide (20 µg/mL, >99%, Carl Roth, Karlsruhe, Germany), and sodium dodecyl sulfate (0.1 mg/mL, >99%, Merck, Darmstadt, Germany).

For the precipitation experiments, castor oil (5 mg/mL, Henry Lamotte Oils, Bremen, Germany), polysorbate 80 (2.5 mg/mL, BioXtra, Merck, Darmstadt, Germany), and Nile Red (8 µg/mL, technical grade, Merck, Darmstadt, Germany) were dissolved in ethanol and filtered through a 200 nm syringe filter (Polypropylene, VWR International, Radnor, PA, USA). The aqueous solution used consisted of deionized water filtered through a 200 nm syringe filter.

The system was purged with a 2% solution of Hellmanex (Hellmanex III, Helma Analytics, Müllheim, Germany) in deionized water, which was filtered through a 200 nm syringe filter (Polypropylene, VWR International, Radnor, PA, USA).

The gas bubble generation and pressurizing of vials was done with nitrogen.

### 2.4. Nanoparticle Characterization and Data Processing

To get additional information about the experiments, the produced particle suspensions were collected and analyzed by a NanoSight NS300 (Malvern Instruments, Malvern, UK) to measure the particle size distribution and concentration. For this measurement, the sample was diluted one thousand times, kept at 25 °C, and illuminated by a laser with a wavelength of 405 nm. The movement of particles was tracked for one minute with a rate of 25 frames per second. Assuming that the viscosity of the liquid corresponds approximately to that of water, the sample was analyzed with a detection limit of 20 arbitrary units. The detected particles were counted to measure the concentration and tracked to measure the Brownian motion, allowing the determination of the size of a single particle.

The data collected by the online detector were analyzed using a Matlab script, which set the baseline to zero by subtracting a low pass filtered version of the signal from itself. The new data track contained the peaks, which exhibited a plateau consisting of single elevated points. Our peak-finding algorithm marked only the highest value of each peak. After identifying the peaks, the script measured the number of peaks per second, the height of the peaks above the baseline, and the width of the peaks at the half height. A fixed value was added to the arbitrary units to compensate an offset induced by the baseline subtraction.

### 2.5. Experiments

Five different nitrogen plug sizes were used in the segmented-flow system to precipitate nanoparticles with different size distributions and concentrations. The liquids were mixed using controlled flow rates of 140 µL/min in the aqueous channel and 60 µL/min in the organic channel. The nitrogen pressure was adjusted to get the aimed plug size. From previous experiments, we knew that the mean particle size generated in plugs of the selected lengths was expected to vary between 95 nm and 170 nm in diameter [24].

The detector was started 5 min after a stable stream was established to allow time for the suspension to reach the detector. The detector measured the particles at 4 min intervals to allow purging of the measurement channel. After three cycles, the measurement was finished and the system was purged to continue with a different plug size.

## 3. Results and Discussion

### 3.1. Particle Concentration

**Figure 2:**
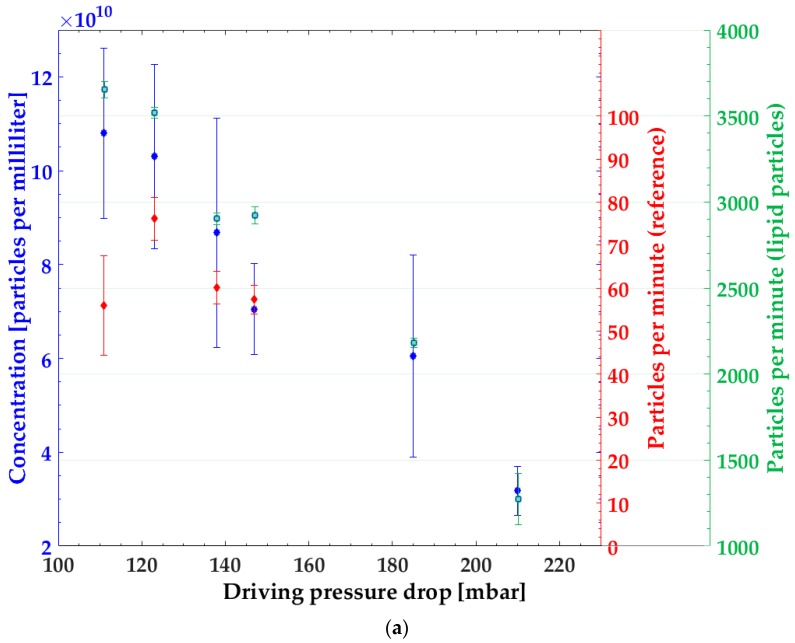

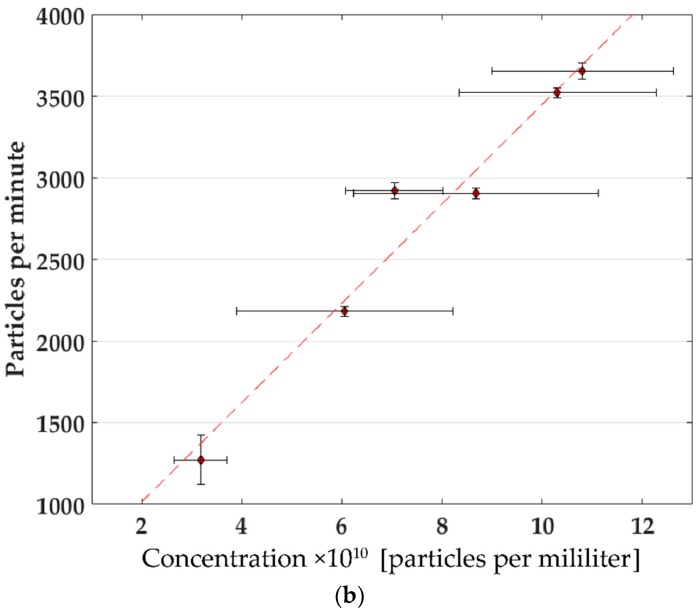
(**a**) Particles per minute measured with the microfluidic detector (red and green) and concentration of lipid nanoparticles in the collected product stream measured by the NanoSight system (blue). Error bars represent the standard errors of the respective measurement techniques. (**b**) Correlation of the microfluidic measured particles per minute and concentration.

First, we investigated the ability to measure the concentration of precipitated nanoparticles continuously using our coupled plug-flow mixer and fluidic bypass detector. The data (Figure 2a) show that by increasing the pressure drop in the synthesis, the concentration of particles in the product stream dropped. Importantly, this drop was consistently measured by our online detection method and off-line by batch measurements with the NanoSight instrument. For the NanoSight measurements, the sample flowing through the bypass was collected, and the concentration of nanoparticles was measured by counting the particles in the volume defined by the objective used in the NanoSight. The concentration of particles measured in the collected product showed the same dependence on the pressure drop as the microfluidic online detection. To find out whether the change in the number of detected particles was related to the change in precipitation or to varied flow velocity, the system was tested with reference particles under the same conditions. In comparison to the precipitated lipid nanoparticles, the reference particles showed no significant change in the number of detected particles with varied flow velocity. The change in particle number was therefore related to the synthesis in the chip and did not depend on changes in flow rates in the setup, which allowed us to correlate the microfluidic measured parameter with the concentration and thus calibrate these parameters for further processes (Figure 2b).

### 3.2. Single-Particle Analysis by Fluorescence

**Figure 3:**
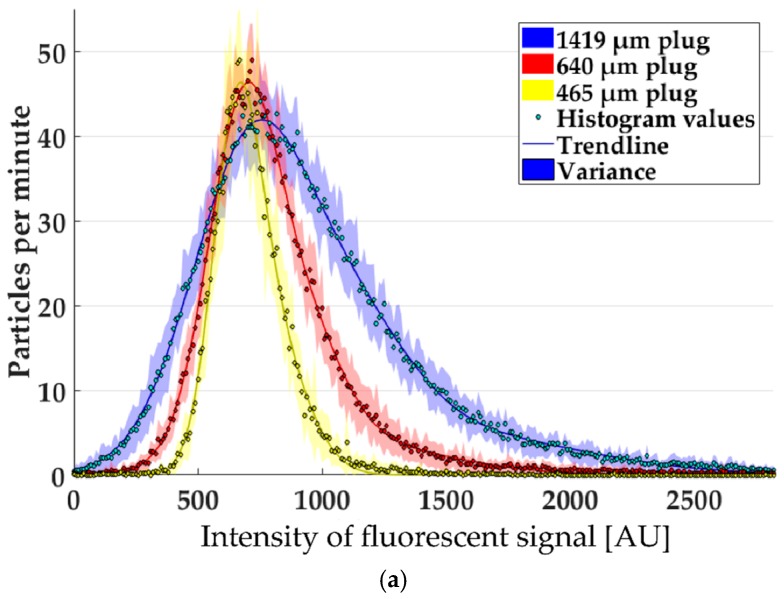

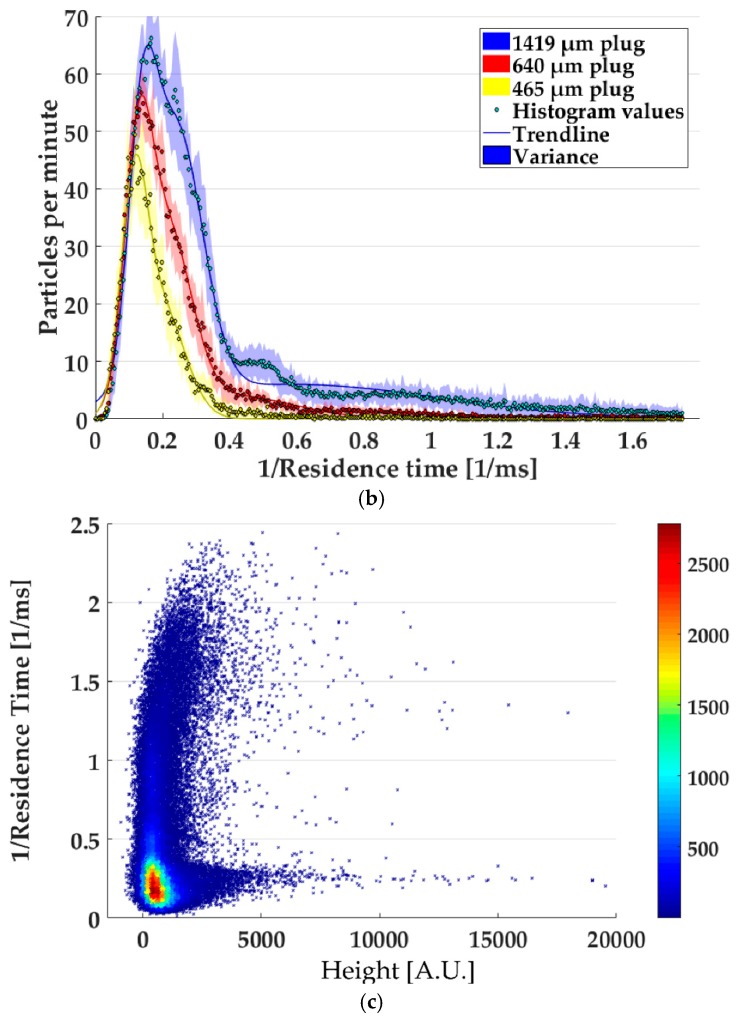
(**a**) Histograms of the fluorescence intensity of different experiments. (**b**) Histograms of the reciprocal residence time of different experiments. (**c**) Heatmap for all particles of a 1419 μm plug experiment to detect correlations between fluorescence intensity and residence time (colored areas in (**a**) and (**b**) represent the variance of three to four repeated experiments under the same conditions).

Given that the concentration of Nile Red in the lipid nanoparticles is uniform, the maximum peak intensities that are detected in our setup are proportional to the volume of the particles. Histograms of the maximum peak intensities measured in experiments with different mixing conditions are shown in Figure 3a. Different mixing conditions were set up by varying the length of the gas plugs between the liquid segments in the mixer.

The histograms of the maximum peak intensities reveal a distribution which can be approximated by a superposition of Gaussian curves. The reduction in plug size leads to a reduction of the mean particle size up to 29%. This reduction is not due to a shift of the maxima in the histograms, but rather due to a narrowing of the distribution, which is more pronounced for particles much bigger than the mean. This change indicates that a bigger plug size leads to an increased polydispersity of the particle suspension.

The histograms of the reciprocal residence time show a very prominent peak in all experiments (Figure 3b). The height of the peaks varies between the experiments. These differences result from the different concentration of particles in each experiment as described before. A change of the mean value between the experiments is detectable, but is not due to a shift of the main peak. Instead, the histograms reveal additional peaks resulting from particles with lower residence times. These peaks are more prominent at larger plug sizes (and a lower pressure drop in the measurement channel) and lead to an increase of the mean value. Since the value represents the reciprocal residence time, this means that some particles were much faster, passing the measurement channel at conditions with a lower pressure drop.

**Figure 4:**
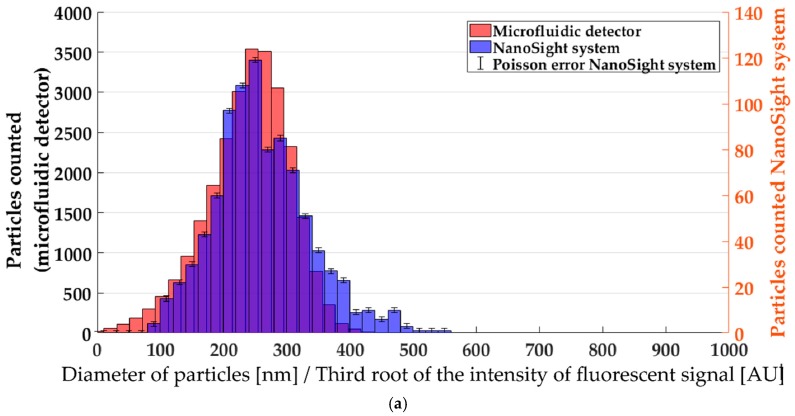

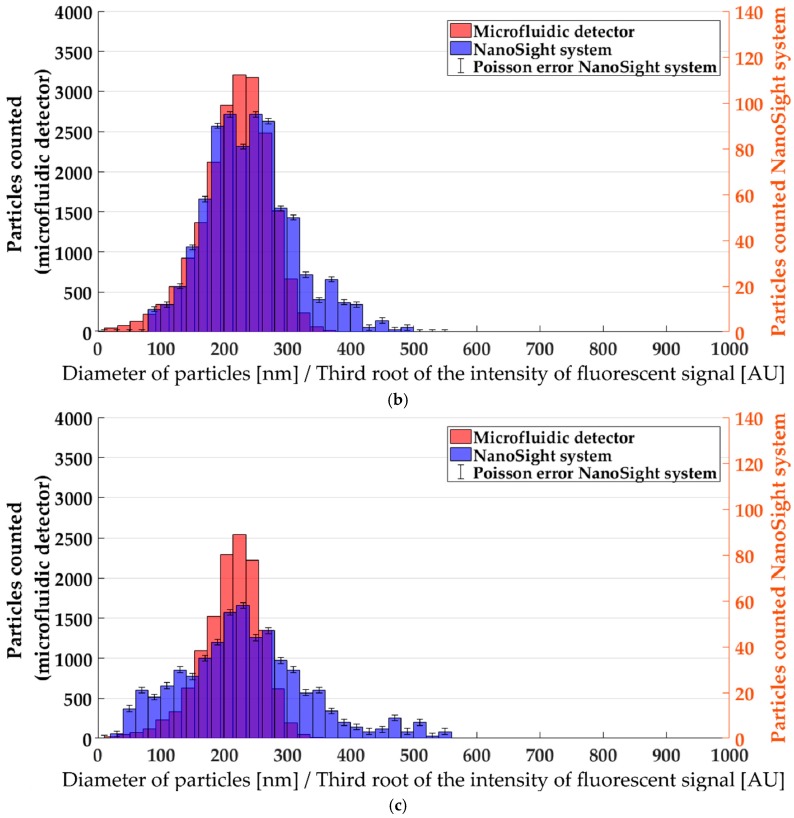
Comparison of qualitative histograms of the third root of fluorescence intensity and particle diameter at different precipitation conditions. The Poisson errors for the microfluidic detector were below a reasonable graphic expression in this context. (**a**) 812 μm plug size. (**b**) 529 μm plug size. (**c**) 386 μm plug size.

The combination of fluorescence peak amplitude and width reveals very interesting details about the particle distribution, as shown in the scatter plot in Figure 4c. While most of the particles follow a narrow size distribution, some particles were scattered over a wider range of sizes and velocities. The interesting point is that the majority of the outliers seemed only to differ in one value from the majority of the points (Figure 3c). This independence of the outliers supports the hypothesis that the phenomena are not connected with each other and even very big particles can move freely in the channel. At a qualitative level, the scatter plot did not reveal any strong indication that outliers in size systematically deviate from the residence time distribution of the majority of the particles. This would be commensurate with the hypothesis that their velocity distribution follows the flow velocity field within the narrow channel. However, a more detailed quantitative analysis would be needed in the future to thoroughly elucidate potential systematic connections between residence time and particle size. Besides the predictable effects of steric hindrance within the small channel, connections between size and residence time distribution could also be suspected for reasons of stiction and other size/morphology-related effects.

To investigate whether the fluorescence intensity histograms measured with the microfluidic device were consistent with nanoparticle tracking analysis (NTA), the sample was collected at the outlet and analyzed with the NanoSight instrument. In this way, it was possible to measure the same sample in the microfluidic detector and by NTA and compare the resulting histograms. In this work, the focus was on measuring relative changes in a given distribution rather than absolute particle sizes. Therefore, the calibration was done by adjusting the arbitrary units on the *x*-axis of the histogram of particle diameters (measured by NTA) and the histogram of the third root of fluorescence intensities (measured by the microfluidic detector). The scale factor for the abscissa was chosen to obtain an overlap at the halved maxima of the two histograms (Figure 4a). The alignment was then transferred on two other measurements (Figure 4b,c) showing that a small shift of the peak maximum between the samples was traceable with both measurement techniques. This made it possible to trace a change in particle size in fluorescence and to establish a connection between particle size and fluorescence intensity. Note that although we consider this calibration sufficient for simple process monitoring, it would be interesting to pursue the option of an absolute independent calibration in future work. This could be accomplished by identifying suitable reference particles whose photophysical characteristics match those of the Nile Red-dyed lipid nanoparticles.

While the peak maximum and the reduction of particle concentration was traceable with both techniques, the polydispersity measured by the NanoSight system was increased in the second and third example (Figure 4b,c). A possible explanation for this is the much smaller number of particles measured with the NanoSight, combined with the possibility of systematic changes in the sample upon collection and transfer to the instrument. Further studies would be needed, however, to investigate this difference in more detail.

### 3.3. Continuous Monitoring to Detect Alterations in Nanoparticle Synthesis

The previous experiments demonstrated the capacity of the setup to detect fluorescent nanoparticles and recognize changes in the process of particle generation, but only under stable process conditions. As a next step, we studied the ability to detect transient situations when the process is destabilized at examples recorded during prolonged experiments previously described.

**Figure 5:**
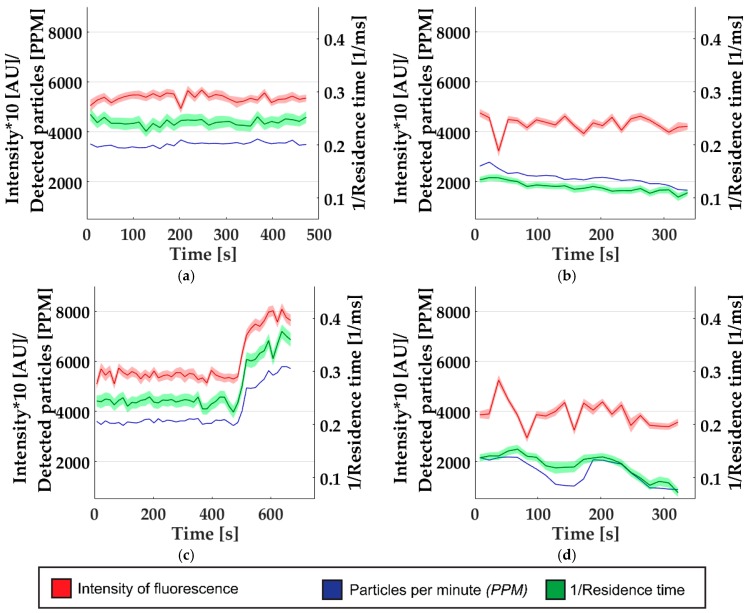
Detected perturbations of the online synthesis. Colored areas represent the standard error of the average value for 15 s measurement frames. (**a**) No incidents. (**b**) Drifting change. (**c**) Incident in synthesis. (**d**) Clogging.

We monitored the signal from the fluorescence detector continuously and analyzed the three measured parameters (single-particle peak amplitude, peak frequency, and residence time) in 15 s intervals. As seen in Figure 5, different types of perturbations could be recorded. The analysis of the three parameters that were recorded allowed a detailed analysis of the transient phenomena. In one experiment, a slow drift in the particle-per-minute count and in the residence time per particle could be detected, as seen in the comparison between Figure 5a,b. This perturbation means that, despite constant synthesis condition, the throughput and velocity in the detector were reduced, which could be the result of fouling. Similarly, a much faster and unstable change in the residence time and the number of particles was detected (Figure 5d), which could be interpreted as clogging of the measurement channel. In both incidents recorded before, the intensity of the particles was not affected by the perturbations, indicating that the precipitation was unaffected. In contrast, in a third detected incident, the precipitation was disrupted, showing changes in all three parameters (Figure 5c). Since the concentration and velocity rose suddenly, a subtle change in flow rate and/or pressure within the fluidic system is likely the cause. A sudden pressure increase can lead to precipitation of larger particles, as seen in the intensity measurement. Using this method, we were able to detect these changes and record the time at which they occurred.

In the future, this information could be used either as a feedback signal to stabilize the synthesis conditions or to suspend the process in time to prevent contamination of the collected product. In such applications, the response time of the detector is an important figure. Our two-chip synthesis/detector system provided interesting insights into realistically attainable response times with similar designs. Here the response time was limited mainly by two factors. First, there was a delay of 90 s in our system until the liquid reaches the detector. Second, at least two data points needed to be collected to identify a step change in the measured parameters. In our example, this added up to a minimum response time of 120 s until a change in synthesis conditions was visible. There is much room for improvement, however, for example by placing the synthesis and detection systems in closer proximity or by increasing the fluidic throughput. Our experience suggests that future systems could realistically be optimized to response times shorter than ten seconds.

## 4. Conclusions

By combining a microfluidic single particle analysis with a particle synthesis chip, we were able to realize an online analysis of nanoparticles synthesized in a microfluidic system. A unique characteristic of our detector is that only a small fraction of the generated particles is withdrawn from the product stream and guided through a detection channel, which has a volume of only 2 pL. Nanoparticles passing through the small channel one-by-one were detected by fluorescence. Transient changes in the particle synthesis were detected in different parameters and could be assigned to a specific time of the process. The single particle signals allow a very detailed analysis based on the histograms and the discussion of outliers.

By detecting the differences between different process conditions, we validated that the stability in size and concentration of the particles—in this process—can be detected, and that incidents happening during the process can be monitored.

To reach a feedback-controlled process, the analysis has to be automated in a single program to allow the fast detection of changes and to counteract those changes by adjusting control parameters of the synthesis (e.g., the driving pressures). Another valuable addition would be a robotic sample collection device that is reached by the stream later than the detector and so could respond to incidents by rerouting the tainted product and protect the already produced particle solution. Since a fast response of such a system is crucial, detection and synthesis should be combined in a single chip to lower the reaction time.

The comparison with particle size measurements, as shown in this work, allow a validation that the changes in intensity are related to a change in particle size. However, for a validated calibration, samples containing the same particles in a narrow size range would have to be measured with the microfluidic detector and an external calibration to correlate intensity to particle size.

In the future, the fundamental limits (e.g., minimum concentration, smallest possible response time) of this approach should also be further studied to allow a better understanding of the applicability of the detector. In addition, by adding static light scattering detection to the setup, the need for a fluorescent dye in the lipid nanoparticles could be eliminated to increase the number of applications for the detector. To increase the long-term stability of the detection, future setups could replace the small measurement channel with a wider channel that incorporates a flow focusing technique. This would increase the resistance of the method against clogging.

In summary, we developed a microfluidic device able to continuously monitor and detect changes in microfluidic nanoparticle synthesis processes, consuming only nanoliter samples.

## Figures and Tables

**Figure A1:**
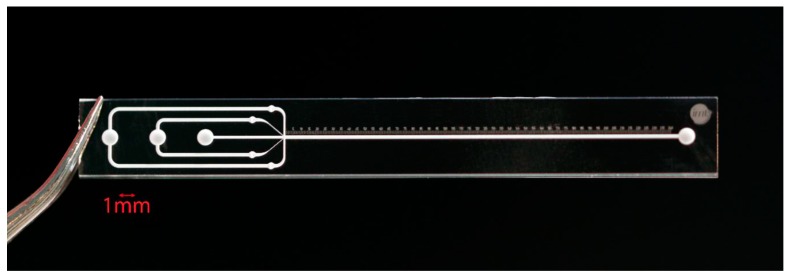
Photograph of the mixer chip

**Figure A2:**
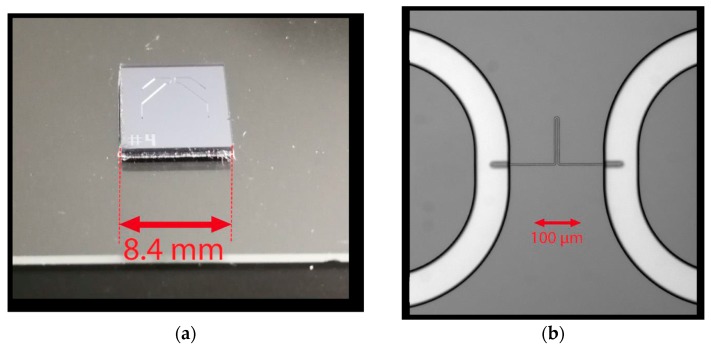
(**a**) Photogrpaph of the detection chip and (**b**) measurement channel.

**Figure A3:**
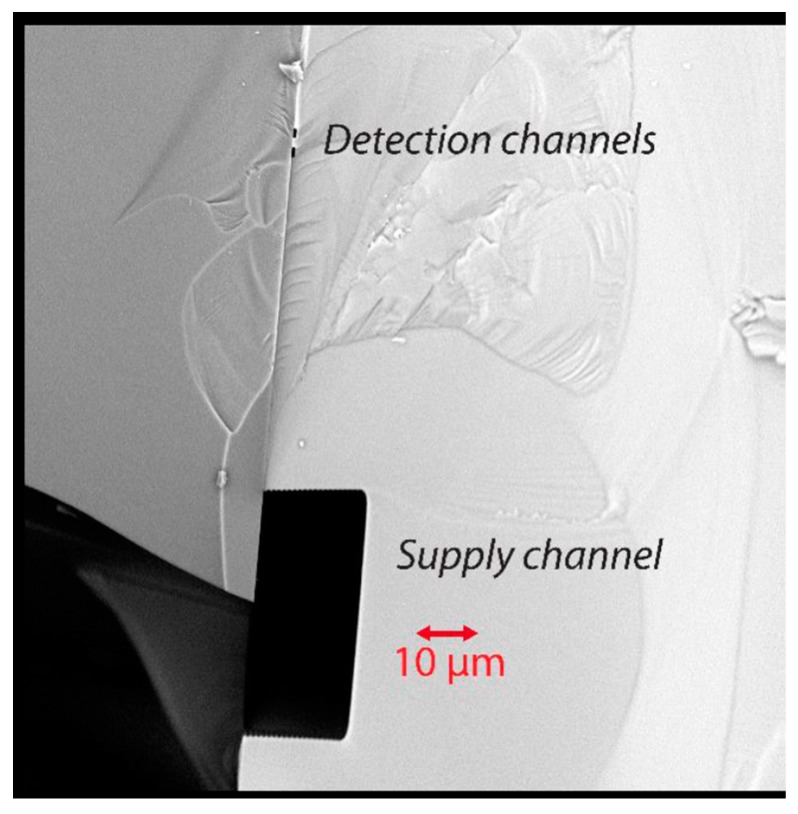
Cross section of channels used in the detection chip.

## Appendix A

### Mixing Chip

The mixing chip is built by combining two glass slides, which are both structured to a depth of 100 μm to achieve a round or oval profile [24]. The fluid channels branch in two symmetrical channels until all streams are combined in a crossing section (Figure A1). To ensure the purity of the liquids, the inlet channels contained particle traps. The crossing section merges five channels into a single stream. In this section the diameter of the different channels vary widely, so that the organic phase containing channels are only 20 μm wide, while the water channels had a width of 100 μm. In addition to these doubled channels the 150 μm wide gas channel is entering the crossing section. The combined stream leaves the crossing section in a straight 2500 μm long and 200 μm wide channel to ensure an optimal mixing process before the liquid leaves the chip.

### Detection Chip

The critical part of the detection chip is the measurement channel with a nominal depth and width of 2 μm. The channel itself has a length of 400 μm, from which 200 μm are shaped in an U to allow the laser alignment without hitting the supply channels (Figure A2b). The supply channels with a width of 75 μm and a depth of 30 μm allow the regulation and exchange of content of the measurement channel by adjusting the pressure between the four access points. To connect the access points by capillary tubings on the back of the chip, the supply channels spread the access points over the area leading to a length of more than 8 mm for each of them (Figure A2a and Figure A3).
